# Effects of natural and seminatural elements on the composition and dispersion of carabid beetles inhabiting an agroecosystem in Northern Italy

**DOI:** 10.1002/ece3.7857

**Published:** 2021-06-29

**Authors:** Francesca Della Rocca, Alfredo Venturo, Pietro Milanesi, Francesco Bracco

**Affiliations:** ^1^ Departement of Earth and Environmental Sciences University of Pavia Pavia Italy; ^2^ Departement of Ecology Czech University of Life Sciences Prague Prague Czech Republic; ^3^ Swiss Ornithological Institute Sempach Switzerland; ^4^ Botanical Garden University of Pavia Pavia Italy

**Keywords:** agricultural landscape, biodiversity, dispersal, line transects, multimodel inference

## Abstract

The natural and seminatural components of agricultural landscapes play a key role in maintaining a high level of biodiversity. Being the Po Valley one of the most human‐dominated and intensively cultivated landscapes in Europe, we investigated the effect of no‐crop habitats on carabid richness and composition and evaluated the role of tree row as corridor for forest carabid dispersion. Carabids were sampled with 70 pitfall traps arranged in 35 sampling plots along three parallel transects (80, 100, and 140 m long) and encompassing five different habitats: tree row, tree row edge, grassland, forest edge, and forest. We found 5,615 individuals belonging to 55 species. Despite the similarity in species richness, all the habitats investigated showed a peculiar and distinct species assemblage. The main distinction was between the "open habitat" cluster composed of grassland and tree row edge and the “forest" cluster composed of forest, tree row, and forest edge. We found that forest species are able to penetrate the grassland matrix up to 30 m from the forest edge and that a distance of no more than 60 m between tree row and forest can allow the passage of up to 50% of the forest species. Beyond this distance, the grassland matrix becomes a barrier, preventing them from reaching other suitable habitats. Our findings confirm the importance of maintaining different types of natural habitats to significantly increase biodiversity in an intensively cultivated agroecosystem and demonstrated the role of linear elements as a corridor and “stepping stones” for many forest species.

## Graphical abstract

Being the Po Valley one of the most human‐dominated and intensively cultivated landscapes in Europe, we investigated the effect of no‐crop habitats on carabid richness and composition and evaluated the role of tree row as corridor for forest carabid dispersion. We found that forest species are able to penetrate the grassland matrix up to 30 m from the forest edge and that a distance of no more than 60 m between tree row and forest can allow the passage of up to 50% of the forest species.

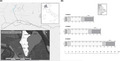

## 1 INTRODUCTION

In the agricultural areas, most of the animal diversity is found in those natural or seminatural features that are not being used primarily for agricultural production (Baudry et al., 2000; Kleijn et al., 2001). These features comprise several types of landscape elements such as wooded patches, uncultivated meadows, marshes, meanders, ditch banks, hedgerows, wooded banks, and small streams. All the natural and seminatural components of the agricultural landscape play a key role in maintaining a high level of biodiversity (Billeter et al., 2008; Davey et al., 2010). They prove important habitat and refuges for rare and endangered species (Ruthsatz & Haber, 1981) and dispersal corridors to support diverse networks of both aquatic and terrestrial taxa. Moreover, they provide enhancement of pollination and biological control as well as functional connectivity within landscapes (Hanley & Wilkins, 2015; Herzon & Helenius, 2008; Marshall & Moonen, 2002).

Wooded and grassland patches are considered among the most stable elements of agroecosystems and important biodiversity hot spots (Herrera et al., 2017; Petit & Usher, 1998). In an intensive agricultural matrix, wooded and grassland patches can be seen as nodes of a network that support the biodiversity found in it (Grashof‐Bokdam & Langevelde, 2004) and favor the flow to and from them by means of linear elements. Linear elements such as hedgerows, wooded banks, and tree rows are arranged around the agricultural fields and form a fine‐meshed network of “veins” (Opdam et al., 2000) that connect the different nodes of the network. Even if they cover a small extension comparing to the crop matrix, they provide functional connectivity to many organisms with different dispersal abilities (McGarigal & Ene, 2012) and provide alternative habitats for species living in a changing environment (Devictor & Jiguet, 2007; Gardner et al., 2007; Hinsley & Bellamy, 2000).

Carabid beetles (Coleoptera: Carabidae) are among the most abundant arthropods of agroecosystems and thought to be an important family of beneficial insects contributing to pest control and acting as a food source for farmland birds (Holland et al., 2005). Carabid beetles have been successfully used as biological indicators of agroecosystem quality (Brandmayr et al., 2005; Cole et al., 2002) because they represent an important component of the epigean terrestrial fauna and reflect the species richness of other orders of insects (Borchard et al., 2014). Moreover, they respond to physical variations in the environment (Brandmayr et al., 2005) and are sensitive to ecosystem alterations due to environmental fragmentation, grazing, fertilization, and deforestation (Rainio & Niemelä, 2003).

The positive effect of spatial landscape heterogeneity on carabid communities inhabiting agroecosystems has recently been demonstrated (Duflot et al., 2016). The presence of forested patches and permanent grasslands connected by linear features of different lengths and compositions is beneficial for them (Duflot et al., 2016; Fahrig et al., 2015; Pecheur et al., 2020; Woodcock et al., 2005) and has been shown to influence both species richness and composition (Schweiger et al., 2005).

The Po Valley, in Northern Italy, is one of the most human‐dominated and intensively cultivated landscapes in Europe (Ingegnoli, 2015). Piedmont and Lombardy regions alone (with about 120,000 ha and 100,000 ha of harvested fields, respectively) contributed to the 52% and 41%, respectively, of the total Italian production (Zampieri et al., 2019). Most of the natural elements of the Po Valley agricultural landscape have almost completely disappeared, and today they persist only within protected areas (Sereni & Litchfield, 1997).

Despite this, to date, few studies explored the effect of no‐crop habitats on carabid richness and composition of the Po Valley agroecosystem (Allegro & Sciaky, 2003; Burgio et al., 2015; Gobbi & Fontaneto, 2008), and only in one case, the role of rice‐field margins as target elements of conservation measures has been investigated (Cardarelli & Bogliani, 2014). Therefore, in the present study, we explored the influence of natural and seminatural features on carabid beetles of the Po Valley agricultural landscape and evaluated the role of tree rows as corridor for forest carabid dispersal.

In particular, we focused on the following aims: (a) to assess differences among carabid beetle communities from different no‐crop habitat in terms of species richness and composition, (b) to identify which environmental factors mostly influence carabid communities, and (c) to define an optimal distance range between wooded patch and linear element that forest carabids can cover moving through inhospitable matrix.

## 2 MATERIALS AND METHODS

### 2.1 Study area

**FIGURE 1:**
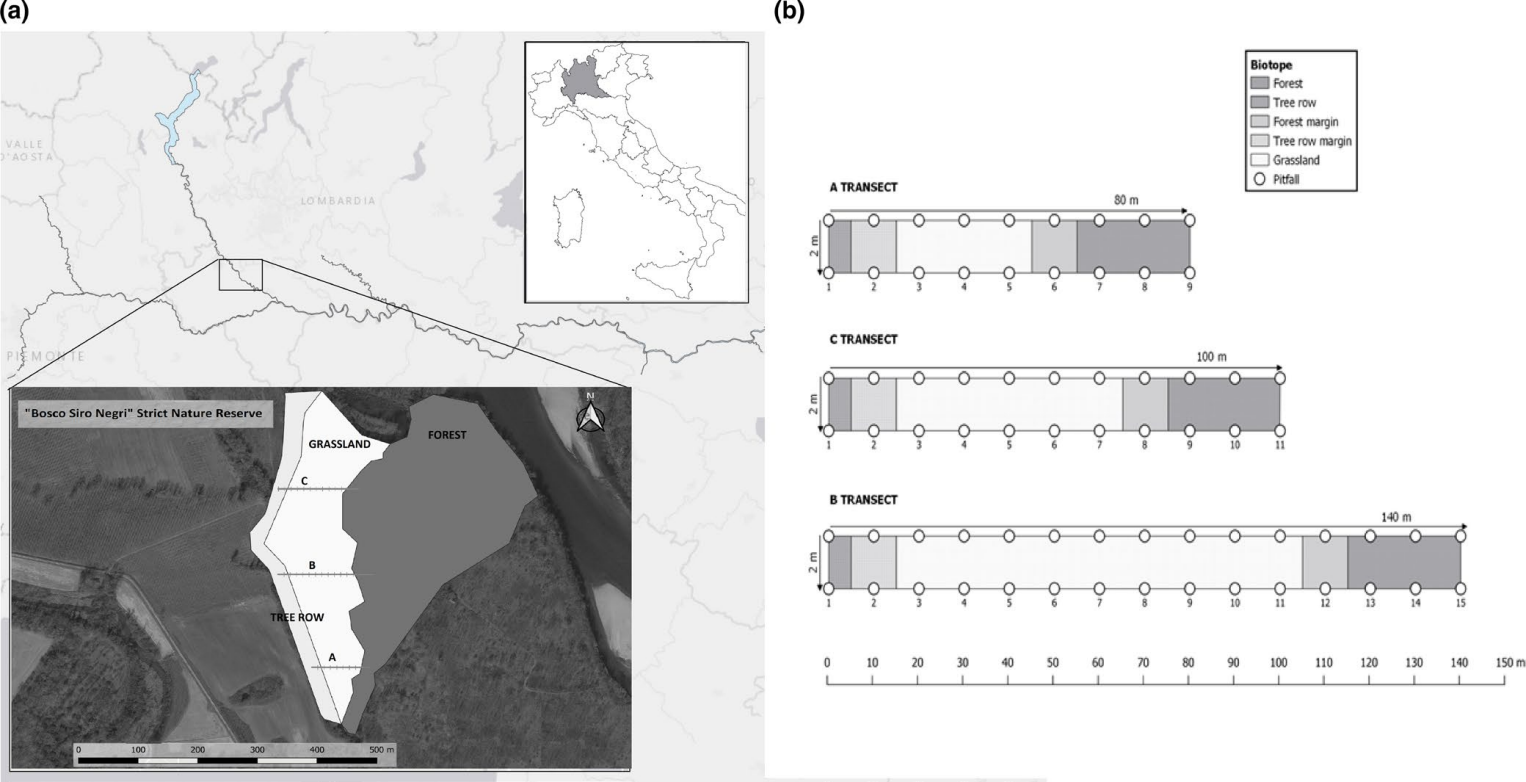
Study area. (a) On top: localization of the study area in Italy and in Pavia Province; on bottom: satellite view of the study area. (b) Scheme of the three transects with highlighted position of pitfalls on each habitat. Each plot consists of a couple of traps separated by 2 m of distance from each other

This study was carried out in the “Bosco Siro Negri” Reserve (45° 12′ 39́” N, 09° 03′ 26′' E; 74 m a.s.l., temperate ecoregion) located on the right bank of the Ticino River, about 15 km from the city of Pavia, in the municipalities of Zerbolò and Torre d'Isola. The reserve belongs to the larger Site of Community Importance IT 2,080,014 "Bosco Siro Negri e Moriano," within the Ticino Valley Regional Park, Italy (Figure 1a). The study area has an extension of about 2.5 km^2^ and consists of a meadow surrounded by mixed deciduous forests on one side and by tree row on the other. The meadow was previously subjected to cultivation but, with the acquisition of the same by the reserve and the consequent interruption of the anthropic disturbance, it is now renaturalizing. The forested area is a relic of the original alluvial forest of Northern Italy (Catoni et al., 2015; Della Rocca et al., 2014) and is characterized by a tree layer dominated by *Quercus robur*, *Robinia pseudoacacia*, *Ulmus minor*, *Populus nigra*, and *Populus alba*, with many of them being more than 100 years old (Castagneri et al., 2013). A subdominant tree layer is characterized by younger individuals of the dominant species and also by *Acer campestre*, *Corylus avellana*, *Prunus padus*, and *Crataegus monogyna*. Due to a high tree density, the forest is characterized by a great light extinction at soil level, but is also interspersed with canopy gaps of variable size (Granata et al., 2016). The forest carabid beetle community inhabiting “Bosco Siro Negri” Reserve was already described by Gobbi et al. (2007), Gobbi and Fontaneto (2008), and Zanella (2013) in her BSc thesis at the University of Pavia, Italy. All these studies reported a high species richness and equitability in species distribution indicating the high ecological value of the “Siro Negri” Reserve forest.

### 2.2 Sampling design and beetle collection

Carabids were sampled with pitfall traps, from 7 June to 28 September 2017 covering a time span with the highest ground beetle activity (Gnetti et al., 2015; Lacasella et al., 2015; Pizzolotto et al., 2014).

We placed a total of 70 traps arranged in 35 sampling plots on three parallel transects along a gradient tree row grassland forest (Figure 1a). Transects had three different lengths (80, 100, and 140 m), were at least 50 m from each other, and spanned the habitat boundary, with 10 m in the tree row, 40 m, 60 m, and 100 m, respectively, in the grassland and 30 m in the forest (Figure 1b). The number of sampling plots per transect varied from 9 to 14 according to the following scheme: 1 plot in the tree row; 1 plot in the row edge; 3, 5, and 9 plots, respectively, in the grassland; 1 plot in the forest edge; and 3 plots in the forest. Within each plot, we placed two pitfall traps, spaced 2 m apart, consisting of 500‐ml plastic cups (90 mm of diameter at the top) filled with 100 ml vinegar, to retain, kill, and preserve individuals (Koivula et al., 2003) and with few drops of soap to break surface tension. Each trap was partially covered with a flat wood roof set approximately at 3 cm above each trap in order to prevent rainwater from entering the trap. All traps were checked continuously for 16 weeks, in order to cover the highest activity of all the species (Gnetti et al., 2015; Lacasella et al., 2015; Pizzolotto et al., 2014), to collect a sample that reflects as much as possible the specific composition of the area (Gobbi et al., 2007; Lacasella et al., 2015), and to meet the recommended standard of 100 days to compensate for random losses (Kotze et al., 2011). Pitfall samples from a plot (two traps) were strained, transferred to 70% ethanol, and pooled together to obtain a single pitfall sample per plot. Captures were later sorted in laboratory, and carabids were separated from other insects and identified to species level by specialists (see acknowledgments) or by comparison with specimens deposited in the entomological collection of the University of Pavia, Italy, by following the nomenclature of *Fauna Europaea web project* (De Jong et al., 2014; www.fauna‐eu.org).

For each species, data on wing development and adult diet were derived from Hůrka (1996), Brandmayr et al. (2005), Homburg et al. (2013) and, when not available from literature, from specialist knowledge. The species have been classified as brachypterous (with reduced wings, not suitable for flight), macropterous (with developed wings, suitable for flight), and dimorphic (with both brachypterous and macropterous individuals) and therefore, respectively, with low, high, and medium dispersal abilities (Brandmayr et al., 2005). As for diet, species were classified as zoophagous, omnivorous, and phytophagous. Wing development and diet provide useful information on the level of disturbance and stability of the environment, with wingless and strictly predatory species negatively affected by human impacts (Gobbi & Fontaneto, 2008; Ribera et al., 2001). Conversely, mobile, omnivorous species are expected to perform better in disturbed and fragmented habitats due to their major dispersal ability and capacity to use different food resources.

For each species, also size, larval development, and habitat preference were recorded following, respectively, Lindroth and Bangsholt (1985), Brandmayr et al. (2005), and Hůrka (1996). According to their size, species have been classified as large (>14 cm), medium (14–6 cm), and small (<6 cm).

### 2.3 Statistical analysis

We used the pooled sample of 70 traps (35 plots) in the analysis. As a measure of species richness, we used the number of species caught in each plot. As a measure of species abundance, we used the number of individuals.

All the statistical analyses were performed on the entire sample of species and on six subsets (zoophagous, phytophagous, brachypterous, macropterous, large species, and medium species) built according to the ecological characteristics described above. Dimorphic, omnivorous, and small species were excluded from the analysis as they consisted of too few species and individuals.

To examine the independence of our plots, we tested our data for autocorrelation by performing a Mantel test based on Pearson's product–moment correlation (permutations: 9,999), between Bray–Curtis distances in assemblage composition and the geographical distances of plots. We found that spatial correlation in assemblages between plots was low (Person's *r* = 0.31) and not significant (*p* > .05). Therefore, we assumed all sampling plots as statistically independent (intersample distance = ≥10 m).

In order to evaluate difference in species richness among transects (transect A: 80 m; transect B: 140 m; transect C: 100 m) and habitats (tree row, tree row edge, grassland, forest edge, and forest), we performed the Kruskal–Wallis test and subsequently the Mann–Whitney post hoc test for pairwise comparisons.

To evaluate difference in species composition among transects and habitats, we performed a PERMANOVA analysis, using the statistical software Primer 6+, with the additional package PERMANOVA + (Anderson et al., 2006; Clarke & Gorley, 2006). PERMANOVA was carried out to test the following null hypothesis (H0): There are no differences, in terms of species composition, between the plots grouped according to the "transect" factor (A, B, and C) and "habitat" factor (tree row, tree row edge, grassland, forest edge, and forest). The analysis was performed on a Bray–Curtis similarity matrix with standardized and square root‐transformed abundance data. Pairwise post hoc comparisons were performed under 9,999 permutations whenever significant differences were found; for further details, see Anderson (2005).

We also used the Bray–Curtis similarity matrix in a principal coordinate analysis (PCO) (Gower, 2005) to display similarities in species composition among all samples. PCO is an unconstrained metric multidimensional scaling ordination that extracts major variance components from the multivariate data set to reduce dimensionality of the data cloud by minimizing the residual variation in the space of any chosen resemblance measure.

To verify whether the diversity in species composition increases with increasing geographic distance (or Euclidean; expressed in meters), we compared the Bray–Curtis ecological dissimilarity matrix with the geographic distance between traps using a linear mixed‐effects model (“lme” function in the “nlme” package in R; Oksanen et al., 2014). The linear mixed‐effects model is generally characterized by three main elements: (a) a response variable, (b) one or more covariates (fixed effects), and (c) a “random” effect. Specifically, we considered the Bray–Curtis ecological dissimilarity matrix as a response variable while the geographic distance matrix between traps as a fixed effect and the Toeplitz covariance matrix as a random effect (Selkoe et al., 2010).

Subsequently, we selected eight environmental variables to characterize the study area both locally and on a landscape level (Table 1). We therefore developed a second linear model with mixed effects considering environmental dissimilarity instead of geographical one, to verify whether the diversity in the species composition is directly proportional to the environmental diversity. Environmental dissimilarity was also estimated using the Bray–Curtis index, but considering the eight environmental variables and their respective values instead of the species detected and their respective abundances. Finally, we carried out a generalized linear model (GLM) to relate species richness and abundance with the eight environmental variables.

**TABLE 1:** Environmental variables selected to characterize the study area at both locally and landscape level

Variable	Description	Mean ± *SD*
Riparian vegetation	Distance from riparian vegetation patches (m)	282.513 ± 37.854
Dense forest	Distance from medium‐density and high‐density broadleaf forest patches (m)	173.933 ± 60.933
Simple crops	Distance from simple crop patches (m)	758.914 ± 79.135
Spare forest	Distance from low‐density broadleaf forest patches (m)	347.681 ± 66.930
Bosco Negri forest	Distance from “Bosco Siro Negri” forest (m)	40.452 ± 42.189
Vegetation cover	Vegetative cover percentage of total area (%)	0.931 ± 0.851
Temperature	Soil temperature measured for each sampling session (°C)	19.936 ± 1.031
Humidity	Soil humidity measured for each sampling session (g/m³)	2.439 ± 0.999

To identify which environmental variables among those selected were most related to species richness, abundance, and composition, we followed the information‐theoretic approach (Anderson et al., 2000, 2001) with multimodel inference. This approach involves the development of as many models (linear in the case of wealth and abundance and mixed in the case of composition) as there are possible combinations between the environmental variables considered (excluding combinations that include correlated variables, |*r*| > 0.7; Dormann et al., 2013). The models obtained were compared using the corrected Akaike information criterion (AICc; Akaike, 1973). The model with the lowest AICc value was selected as the “best” model and, to order the subsequent models, the difference (ΔAICc) between the AICc of the best model and that of the other models was calculated. Furthermore, the Akaike weight (*wi*) was then calculated for each model; this value can be interpreted as the probability of a given model being the best among all those considered (Akaike, 1981). Following the indications of Anderson and Burnham (2002), in addition to the best model (ΔAICc = 0), we considered all the models with ΔAICc < 2.

Finally, to evaluate the dispersal ability of forest species through the inhospitable matrix represented by the grassland habitat, we analyzed the subsample consisting of the 14 forest species and evaluated the relationship between the number of forest species and the distance from the forest edge using a linear model with a second‐order polynomial function fit to the model.

## 3 RESULTS

### 3.1 Carabid beetle richness, abundance, and composition

We collected a total of 5,615 individuals belonging to 55 species (14 of which are forest carabids, Table S1). For each plot, we collected an average of 15.4 ± 3.5 species and 160.4 ± 117.8 individuals. The most abundant species was *Poecilus versicolor*, which represent 21.5% of the total, followed by *Calathus* *melanocephalus* (13.8%), *Calathus* *fuscipes* (10.1%), *Carabus convexus* (8.7%), *Abax continuus* (7.6%), *Calathus* *rubripes* (7.6%), *Pterostichus melanarius* (5.7%), *Pseudoophonus rufipes* (5.6%), *Limodromus assimilis* (4.3%), *Metallina lampros* (2.6%), *Synuchus vivalis* (2%), *Carabus granulatus* (1.3%), and *Harpalus tardus* (1.2%). The residual 76.4% of sampled species had a percentage frequency smaller than 1%. Most of the species collected were macropterous (76.4%), with medium body size (76.4%) and typical of open habitat (49.1%). More than 60% of the species sampled were zoophagous (61.8%) with summer larvae development (65.5%).

Species richness and abundance resulted significantly different among transects (richness: Kruskal–Wallis chi‐squared = 8,728, *df* = 2, *p*‐value = .013; abundance: Kruskal–Wallis chi‐squared = 11,162, *df* = 2, *p*‐value = .004) with the highest mean number of species and individuals in the transect A (Mann–Whitney post hoc test: A versus B, *p* = .038; A versus C *p* = .015; B versus C, *p* = .774) (Table 2). Carabid richness and abundance were similar among habitats (richness: Kruskal–Wallis chi‐squared = 6.8413, *df* = 4, *p*‐value = .144; abundance: Kruskal–Wallis chi‐squared 9.099, *df* = 4, *p*‐value = .058) (Table 2). However, in the forest we found a significantly higher mean number of zoophagous, brachypterous, and large species compared with those found in the grassland, while in the latter, we found a significantly higher mean number of phytophagous species (Table S2).

**TABLE 2:** Mean number of carabid species and individuals per plot collected in each transect and habitat

Categories	No. species		No. individuals	
	Mean/plot	*SD*	Mean/plot	*SD*
Transects				
A	18.3	±3.0	269.4	±146.1
B	14.3	±3.8	132.5	±93.8
C	14.5	±2.1	109.4	±55.5
Forest	6.1	±3.0	28.8	±28
Forest edge	6.5	±2.7	22.9	±14.6
Habitats				
Grassland	4	±3.0	16.4	±23.9
Tree row margin	4.9	±3.2	22.8	±42.0
Tree row	5.8	±3.5	19.9	±24.2

PERMANOVA analysis showed that species composition differed significantly among habitats but not among transects (p (MC) = 0.564; *Pseudo*‐*F* = 0.880, *df* = 2; *p* (MC) < .0001; *Pseudo*‐*F* = 4.346, *df* = 4, respectively). The post hoc test revealed that most of the habitats analyzed differed significantly from each other in terms of species composition. Forest species assemblage significantly differed from that of grassland, forest edge, tree row, and tree row edge (forest versus grassland: *t* = 3.505, *p* < .001; forest versus tree row edges: *t* = 2.876, *p* = .003; forest versus forest edge: *t* = 1.832, *p* = .033; forest versus tree rows: *t* = 1.719, *p* = .059). Grassland species assemblage differed from that of tree row and forest edge (grassland versus tree row: *t* = 1.919, *p* = .008; grassland versus forest edge: *t* = 1.567, *p* = .038), other than from that of forest as mentioned above, but did not differ from that of tree row edge (grassland versus tree row edge: *t* = 1.181, *p* = .215). Species composition differed significantly among habitats for all the subset of carabids analyzed with the exception of phytophagous species (Table S3). In particular, for all the subsets, a statistically significant difference emerged between grassland and tree row, between grassland and forest, and between tree row edge and forest (Table S4). Zoophagous and brachypterous species composition differed also between tree row and forest and between grassland and forest edge while zoophagous and macropterus species composition differed also between forest and forest edge (Table S4).

**FIGURE 2:**
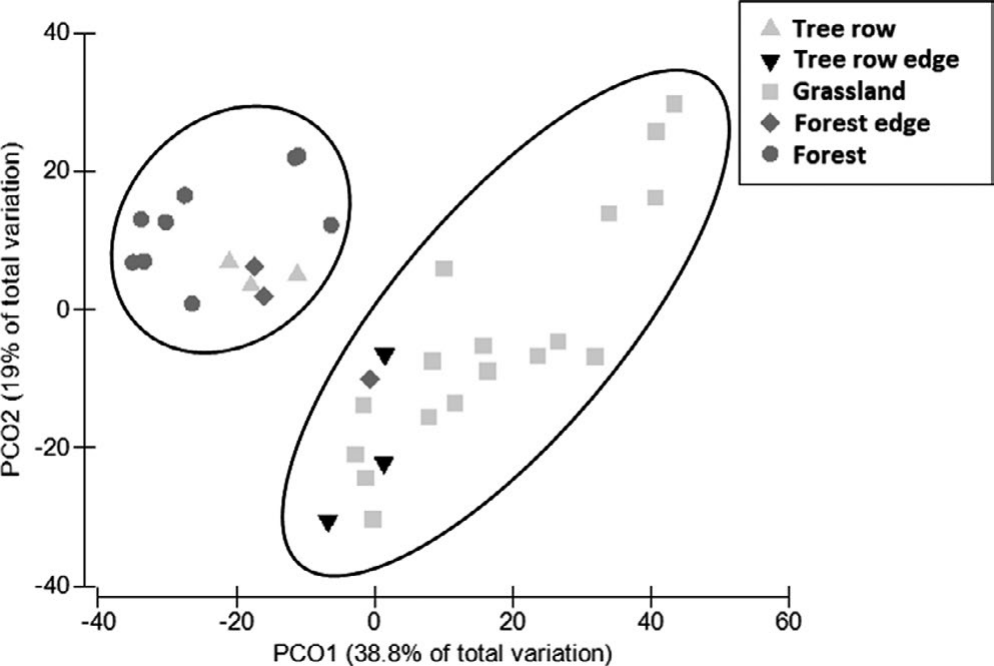
Scatter plot showing the ordination produced by principal coordinate analysis (PCO) in the Bray–Curtis distance matrix for the 35 plots belonging to 6 habitats. Each trap is ordered in space on the basis of the degree of similarity that exists with the other plots. Along the first axis, which explains the 38.8% of total variation, it is possible to identify two groups: one group composed of forests, forest edges, and tree row plots; the other group composed of grassland and tree row edge plots

Principal component analysis (PCO) aggregates all the plots in two main similarity clusters along the first axis (38.8% of variance): one group including plots from forest and tree row and one group including tree row edge and grassland (Figure 2).

### 3.2 Factors affecting carabid species richness and composition

Multimodel inference for species richness showed that the best predictor models with AICc < 2 included from one to five factors (Tables 3, S5 and S6). Considering all the models, humidity resulted as the most relevant factor which positively influenced both species richness and abundance. All the other factors are significant for species richness but not for abundance and have a considerably lower relative importance.

**TABLE 3:** Effect of environmental variables on the richness and abundance of carabid species present in the study area obtained from multimodel inference

Covariates		Richness	Abundance
Humidity	*β*	**1.640**	**59.839**
	*Ri*	**0.261**	**0.249**
Simple crops	*β*	**−0.020**	−0.549
	*Ri*	**0.261**	0.183
Riparian vegetation	*β*	**−0.038**	−0.454
	*Ri*	**0.261**	0.145
Spare forests	*β*	**−0.017**	4.514
	*Ri*	**0.106**	0.047
Bosco Negri Forest	*β*	**−0.023**	−0.522
	*Ri*	**0.113**	0.134
Temperature	*β*	‐	−0.043
	*Ri*	‐	0.059

Note

The table shows the estimates of the standardized mean regression coefficients (*β*) and the relative importance (*Ri*) of each environmental variable. The relative importance (*Ri*) was calculated as the importance (sum of the "Akaike weights" of all models) of a given variable divided by the sum of the importance of all variables for each subset of data. The dashes indicate that the term considered does not appear in the best model set. Variables whose *β* confidence interval does not include 0 can be considered to have a significant effect (values expressed in bold).

Multimodel inference for species composition led to the development of 256 mixed‐effect linear model. For each model, we calculated AICc, and only one of these models resulted to have ΔAICc < 2 (Table 4). Such model takes into account four environmental factors: vegetative cover, distance from Siro Negri forest, distance from crops, and humidity. All these factors were significantly and positively correlated with species composition (Table 4).

**TABLE 4:** Results of multimodel inference conducted using mixed‐effect models. Only models with ΔAICc <2 are shown

Covariates	*β*	*P*
Intercept	40.831	<.0001
Vegetation cover	0.122	<.0001
Bosco Negri Forest	0.073	<.0001
Simple crops	0.791	<.0001
Humidity	0.346	<.0001
*df*	7	
logLik	−2.354	
AICc	4,722	
*R* ^2^ Marginal	0.357	
*R* ^2^ Conditional	0.357	

### 3.3 Dispersal capacity of forest species through inhospitable matrix

**FIGURE 3:**
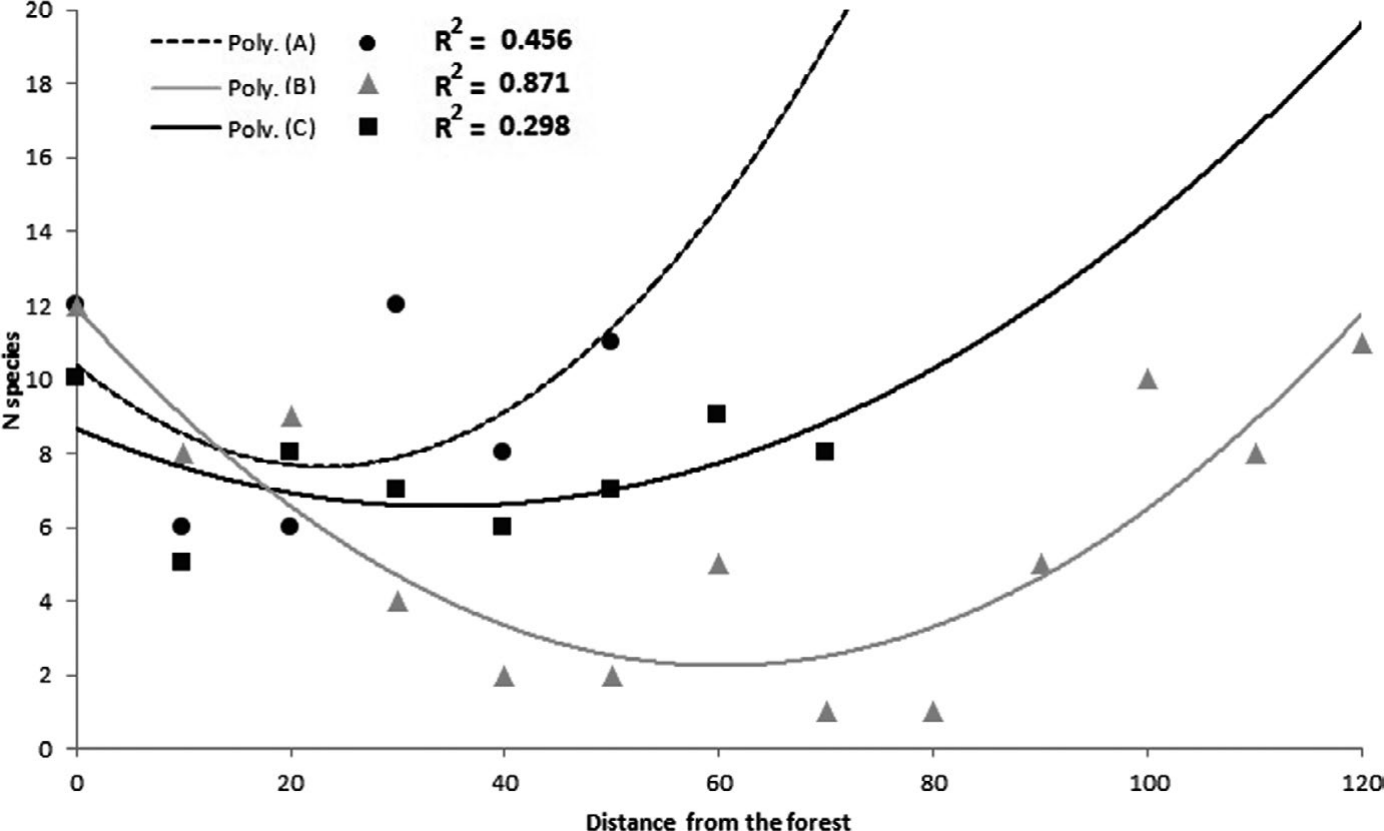
Number of species in each transect as a function of the distance from the forest edge. On the right side of the graphic, the number of species increased as the proximity to the tree row increases. The tree row is located at a distance from forest edge of 40 m for transect A, 90 m for transect B, and 50 m for transect C. A linear model with a second‐order polynomial function was fitted to the model. For each trend line, its relative R2 is shown

Forest species richness decreased with the increasing of the distance from forest edge until it reached a negative peak in the middle of grassland matrix after which it began to rise again as the distance from the tree row decreases (Figure 3). On transect A, the number of species reached the minimum value (with a decrease of 36.3% from the initial value) in the plot located at 20 m from the forest edge and 20 m from the tree row edge. Most of the species found in this plot (6 out of 7) were shared with both the forest and tree row (Table 5). Moreover, most of the species collected in the tree row plot were also collected in forest plots (12 out of 14).

**TABLE 5:** Distribution of forest species along transects A, B, and C

Species	Forest			Inhospitable matrix			Tree row		
	A	B	C	A	B	C	A	B	C
*Abax continuus*	36	225	58	2	0	3	3	8	7
*Calathus rubripes*	76	183	103	0	0	0	6	7	3
*Carabus convexus*	84	32	45	2	0	0	45	51	34
*Carabus granulatus*	5	32	13	0	0	0	3	2	6
*Limodromus assimilis*	66	89	40	0	0	0	19	8	1
*Limodromus krynickii*	2	12	7	0	0	0	12	0	1
*Patrobus atrorufus*	2	6	0	0	0	0	5	0	0
*Pterostichus melanarius*	9	58	21	1	1	5	18	24	14
*Pterostichus niger*	6	13	4	0	0	0	0	3	1
*Pterostichus strenuus*	5	1	1	0	0	1	1	1	0
*Pterostichus vernalis*	0	0	0	1	0	1	0	0	0
*Stomis pumicatus*	3	3	0	1	1	2	2	1	0
*Synuchus vivalis*	4	5	3	1	0	0	18	1	0
*Nebria brevicollis*	0	0	0	0	0	1	0	0	0

Note

The number of specimens of each species is shown. The first and last columns represent the forests and tree row plots while the central column (Inhospitable matrix) represents the plot located in the middle of grassland where the minimum number of forest species has been collected.

On transect C, the number of species reached the minimum value (with a decrease of 45.5% of the initial value) in the plot located at 40 m from the forest edge and 20 m from the tree row edge. In this plot, four out of seven species were shared with both the forest and tree row (Table 5). Moreover, most of the species collected in the tree row plot were also collected in forest plots (nine out of 11). The lowest number of forest species was found in transect B in the plots located at 70 and 80 m from the forest edge and 30 from the tree row edge. Here, we collected only two forest species (8% of the initial value), *Pterostichus melanarius* and *Stomis pumicatus*, also present in both forest and tree row (Table 5). Most of the species collected in this transect (11 out of 15) were shared between forest and tree row.

## 4 DISCUSSION

In this study, we analyzed the role of adjacent natural and seminatural habitats for carabid assemblages inhabiting an intensively managed agroecosystem. Our results highlighted the existence of high difference in species richness and assemblage composition among adjacent habitats. Our results further demonstrate the role of linear element in forest grassland mosaic as corridor for forest carabids species and the importance of its distance from the forest in shaping the carabids species assemblage inhabiting the adjacent open habitats.

### 4.1 Carabids species assemblages

The current composition of the carabids community inhabiting the “Bosco Siro Negri” Reserve reflects the heterogeneity of the investigated agricultural landscape. Most of the sampled species (50%) are simultaneously macropterous and predators (*Poecilus versicolor* and *Calathus* *fuscipes* are the most abundant) with great potential for natural pest control (Holland & Luff, 2000; Symondson et al., 2002; Tscharntke et al., 2012). They usually inhabit open areas, such as grasslands or crops, and are typical of unstable and entropized environments (Brandmayr, 1983). In most cases, they have summer larval development, which is an opportunistic reproductive adaptation, and limited body dimension favored by the presence of disturbed environments (Blake et al., 1996). However, most of the macropterous species collected in this study were extremely low abundant compared with the brachypterous ones. The former, mainly collected in forest habitat, are typical of more stable environments (Brandmayr et al., 2005; Kotze et al., 2011) and indicate an improvement of the agricultural landscape and the expanding woodland patches from the past to today as reported by Koivula et al. (2002), Poole (2002) and specifically for Lombardy's lowland areas by Sartori and Bracco (2012). The high ecological value of the “Siro Negri” Reserve forest is confirmed by the presence of two endemic beetles: *Abax continuus* and *Calathus* *rubripes* (Gobbi et al., 2007) already found in previous monitoring studies carried out in the reserve (Gobbi et al., 2007; Zanella, 2013). Because of their low dispersal capacity, the persistence of high abundances of these species in the studied area demonstrates the existence of a very stable forest environment and the effectiveness of forested linear elements, such as that under study, as ecological corridors for them.

Our study showed that carabid beetle composition varies among habitats in a more or less marked manner according to the different degrees of adaptation of the species to their habitat. The main distinction was between the “open habitat” cluster composed mainly of samples from grassland and tree row edge, and the “forest" cluster composed mainly of samples from forest, tree row, and forest edge.

The “open habitat” cluster is characterized mainly by macropterous beetles with medium body size and phytophagous diet, typical of open habitat (Brandmayr et al., 2005; Gobbi & Fontaneto, 2008). These species are unable to penetrate wooded patches within a certain extent; therefore, their presence gives the grassland habitats a unique specific composition (Lacasella et al., 2015; Magura et al., 2017; Niemelä, 2001). Some forest predator species, due to their greater capacity to penetrate inhospitable environments (Lacasella et al., 2015; Magura et al., 2017; Niemelä, 2001), were also found in this habitat. Despite their lower abundance, the presence of predator species favored by the trees shading of the surrounding forest habitats (both linear element and patches) is fundamental in providing more stable habitat condition and in ensuring a natural pest control function (Symondson et al., 2002; Tscharntke et al., 2012).

The “forest” cluster is characterized by a high number of species. Most of them are hygrophilous (half of which brachypterous) suitable for living in high soil humidity conditions and highly linked to forest habitat. However, we sampled also several generalist species, mostly found in forest edge and tree row samples, able to move in suboptimal environments and to penetrate the grassland matrix to a certain extent (Niemelä, 2001). Few grassland‐associated species are present because of their inability to penetrate in forest (Lacasella et al., 2015). Humidity was found to be one of the parameters that most influence the specific composition of the carabid community under study. Humidity is likely to act selectively on carabids at the first‐larval stages when the weak chitinization and limited mobility make them more sensitive to desiccation (Lövei & Sunderland, 1996). Different studies have pointed out humidity as a key factor shaping soil arthropod distribution (Bogyó et al., 2015; David & Handa, 2010; Pearce & Venier, 2006) especially in old riparian forests where many drought‐sensitive species are present (Baker et al., 2019). Because humidity is lower at forest edges compared with interiors (Chen et al., 1995; Gehlhausen et al., 2000), the different species compositions observed between forests samples from one hand and tree rows and forest edge from the other hand are probably due to the different abilities of the species to withstand dry conditions and persist in forest edges compared with drought‐sensitive species that retreat to forest interiors (De Smedt et al., 2018). Moreover, as Brandmayr (1991) pointed out, hydric conditions affect the dispersal power of carabid beetles favoring an increasing of brachypterous species in those environments with a more stable soil water balance and a reduced inundation risk. Also, the well‐documented spillover from adjacent fields could be considered an important cause of compositional variation between more exposed habitat (such as forest edges and tree row) and the forest interiors (Boetzl et al., 2016; Tscharntke et al., 2012). In our case, this phenomenon can be generally considered of minor concern, because, as also observed by previous studies (Lacasella et al., 2015; Magura et al., 2017), grassland‐associated species are more sensitive to edge effect and influence forest community less than the reverse. However, the different nature of the two habitats, forest edge and tree row, should be considered. The first is interposed between two different habitats, forest and grassland, and has a stratified horizontal structure composed of shrub and sapling zone toward the forest interior, and a perennial herb layer toward the adjacent open habitat (Forman & Godron, 1986). The second, on the other hand, although connected to the forest, is interposed between two open habitats (in the specific case of this study, grassland and crop field) and has a simplified vegetation structure and generally reduced habitat heterogeneity (Forman & Baudry, 1984). Therefore, the different sets of environmental conditions that characterize the two habitats would impose different environmental filtering and select related species (also coming from adjacent habitat) with specific traits that allow coping with these specific habitat conditions (Magura & Lövei, 2019). This will result in two unique and distinct elements of the agroecosystems, each with its own peculiar specific composition that should not be considered merely the sum of the two adjacent habitats, but an emergent property of the forest–grassland interface (Lacasella et al., 2015). The unique species composition of tree rows is also confirmed by Lovei & Magura (2017) who described a rich ground beetle community inhabiting the edges of Danish agricultural landscape.

We have already described the importance of humidity as the main factor in driving species richness, abundance, and composition of the forest carabid communities of the Bosco Siro Negri Nature Reserve. This parameter seems to be decisive also in affecting phytophagous species generally more adapted to live in open habitat with low soil moisture (Brandmayr et al., 2005; Holland, 2002; Kromp, 1999; Lövei & Sunderland, 1996). Other important factors affecting the carabids community under study were the distance from some elements of the agricultural landscape such as simple arable land, broad‐leaved woods, and riparian vegetation patches, while factors such as temperature are less significant. The importance of forest patches (Rainio & Niemelä, 2003), arable land (Kromp, 1989; Pavuk et al., 1997), and also the simultaneous presence of both has already been recognized for carabids (Armstrong & McKinlay, 1997). The increase in diversity and abundance of carabids in relation to the distance from some elements of the landscape could also be due to a general increase in complexity in the vegetative structure, a factor also considered positive for the presence of carabids (Fournier & Loreau, 1999; Holland, 2002). Another important factor affecting abundance and specific composition is the vegetative cover. In particular, as already highlighted by Jopp and Reuter (2005), it seems that an excessively high density of vegetation is an impediment to the gait of carabids, especially for larger and wingless species.

### 4.2 Dispersal capacity of forest species through inhospitable matrix

In this study, about 70% of the overall forest species already described for the Bosco Siro Negri Reserve (Gobbi et al., 2007; Zanella, 2013) have been collected. Therefore, although the extension of the transect stopped about 30 meters from the edge and do not reach the innermost part of the forest, the forest species sampled can be considered representative of the entire community of forest carabids inhabiting the Bosco Siro Negri Reserve.

Several studies investigated the agroecosystem arthropod community response to the two‐sided edge effects at the forest–grassland ecotone quantifying the mutual influences of two adjacent terrestrial habitats on carabid species abundances and distributions (Lacasella et al., 2015; Roume et al., 2011). A much greater number of studies investigated the role of linear elements on the distribution of carabid beetles in agroecosystems (Gallé et al., 2019; Petit & Usher, 1998) and the effects of forest carabid dispersal on the adjacent crop field species assemblages (Moerkens et al., 2010). However, to date no studies have taken into consideration the simultaneous effect of two different forest habitats (linear and patch) on the carabid community inhabiting the encompassed grassland matrix.

Based on what is currently known, forest carabid beetles were able to enter the grassland influencing its specific composition up to a distance of about 5–20 m depending on the different methodological designs adopted and the biogeographical and ecological context in which they were conducted (Bedford & Usher, 1994; Bieringer et al., 2013; Heliölä et al., 2001; Lacasella et al., 2015; Magura, 2002).

In our study, we found that the dispersal capacity of many forest species is greater, allowing them to penetrate the grassland matrix, altering its composition, up to 30 meters from the forest edge. Moreover, if a tree row bordering the grassland is present, the number of forest species able to cross the matrix will increase with the decreasing of the distance to this forested linear element. In the case of the Bosco Siro Negri Reserve, a distance of no more than 60 m from the tree row and the forest can allow the passage of up to 50% of the forest species. Beyond this distance, the grassland matrix becomes a dispersive barrier for these beetles, preventing them from reaching other suitable habitats.

## 5 CONCLUSIONS

For a sustainable management of the intensively cultivated agroecosystem, the structural heterogeneity of the fields should be increased by the implementation of natural and seminatural elements (Holland et al., 2005; Pecheur et al., 2020). Indeed, different types of habitats with their own peculiar species composition contribute to significantly increase biodiversity (Holland et al., 2005; Tsonkova et al., 2014) by providing adjacent habitats with continuous species flow. The presence of hedges and rows in the agricultural landscape contribute to significantly increase the forest species within the crop fields with beneficial effect on the agriculture because most of them are known to prey on pests (Symondson et al., 2002). Our findings also show the potential role of linear elements as “stepping stones” for many forest species favoring their movement from one forest patch to another through the agricultural matrix and could provide a measure of the distance between patches at which this movement can occur. We found that grassland habitat has unique species composition. The importance of these habitats, generally considered of secondary conservation value (Bond & Parr, 2010; Bremer & Farley, 2010; Willis & Bhagwat, 2010), has been recently re‐evaluated by demonstrating the existence of many peculiar associated species (Lacasella et al., 2015; Pawson et al., 2010; Tab oada et al., 2004). Therefore, an excessive presence of forested patches surrounding grasslands and a high number of species coming from adjacent environments could alter the uniqueness of these habitats (Lacasella et al., 2015) and overestimates their value within mosaics for biodiversity conservation.

Therefore, because the conservation of biodiversity in a complex landscapes such as the agricultural one will depend on the ability to preserve both forest and open habitats within the landscape, an accurate territorial planning should always take into account the reciprocal effects of these adjacent habitats and the spatial extent of their interactions.

## CONFLICT OF INTEREST

None declared.

## AUTHOR CONTRIBUTIONS

**Francesca Della Rocca:** Conceptualization (lead); Data curation (lead); Formal analysis (equal); Investigation (lead); Methodology (equal); Project administration (lead); Supervision (lead); Writing‐original draft (lead); Writing‐review & editing (equal). **Alfredo Venturo:** Data curation (equal); Investigation (equal); Writing‐review & editing (equal). **Pietro Milanesi:** Formal analysis (equal); Methodology (equal); Software (equal); Writing‐review & editing (equal). **Francesco Bracco:** Funding acquisition (lead); Resources (lead); Writing‐review & editing (equal).

## Supporting information

Table S1‐S6

Appendix S1

## Data Availability

Species data collected in this study are available in Appendix S1 (excel file).
