# Supplementary material for: Effects of natural and seminatural elements on the composition and dispersion of carabid beetles inhabiting an agroecosystem in Northern Italy
Source: Ecol Evol. 2021 Jun 29;11(15):10526–37. doi: 10.1002/ece3.7857 (PMC8328445; doi:10.1002/ece3.7857)
Supplement: Supplementary file 1 — Table S1‐S6 [file ECE3-11-10526-s001.docx]

**SUPPLEMENTARY MATERIAL**

**Tab. S1.** List of sampled species with the relative number of specimens (N) and some of the main ecological characteristics: Wing morphology (B = brachypetrous, M = macropterous, D = dimorphic), Diet (ZOO = zoophagous, OM = omnivorous, PHY= phytophagous), Size (SMA= small, MED = medium, LAR = large), Larval development (SUM = summer, WIN = winter, ANN = annual), Habitat preference (F = forest, O = open habitat, W= wetland, MH= More habitat)

| SPECIE | N | WING | DIET | SIZE | LARVA | HABITAT |
| --- | --- | --- | --- | --- | --- | --- |
| *Abax continuus* | 426 | B | ZOO | LAR | ANN | F |
| *Agonum afrum* | 24 | M | ZOO | MED | SUM | W |
| *Agonum sexpunctatum* | 2 | M | ZOO | MED | SUM | W |
| *Amara aenea* | 4 | M | OM | MED | SUM | O |
| *Amara convexior* | 5 | M | OM | MED | SUM | O |
| *Amara lucida* | 1 | M | OM | MED | SUM | O |
| *Anchomenus dorsalis* | 3 | M | ZOO | MED | SUM | O |
| *Anisodactylus binotatus* | 4 | M | OM | MED | SUM | W |
| *Anisodactylus nemorivagus* | 1 | M | OM | MED | SUM | W |
| *Anisodactylus signatus* | 1 | M | OM | MED | SUM | W |
| *Asaphidion flavipes* | 4 | M | ZOO | SMA | SUM | W |
| *Badister bullatus* | 3 | M | ZOO | MED | SUM | W |
| *Bembidion quadrimaculatum* | 1 | M | OM | SMA | SUM | O |
| *Brachinus elegans* | 1 | M | ZOO | MED | SUM | O |
| *Brachinus explodens* | 1 | M | ZOO | MED | SUM | O |
| *Calathus fuscipes* | 566 | M | ZOO | MED | WIN | O |
| *Calathus melanocephalus* | 772 | B | ZOO | MED | WIN | O |
| *Calathus rubripes* | 425 | B | ZOO | MED | WIN | F |
| *Carabus convexus* | 487 | B | ZOO | LAR | SUM | F |
| *Carabus granulatus* | 71 | B | ZOO | LAR | SUM | F |
| *Chlaeniellus nitidulus* | 2 | M | ZOO | MED | SUM | W |
| *Dolichus halensis* | 2 | B | ZOO | MED | WIN | O |
| *Dromius linearis* | 3 | M | ZOO | SMA | SUM | W |
| *Drypta dentata* | 1 | M | ZOO | MED | SUM | W |
| *Harpalus anxius* | 12 | M | PHY | MED | SUM | O |
| *Harpalus dimidiatus* | 17 | M | PHY | MED | SUM | O |
| *Harpalus pumilus* | 1 | M | PHY | MED | SUM | O |
| *Harpalus rufipalpis* | 18 | M | PHY | MED | SUM | O |
| *Harpalus serripes* | 36 | M | PHY | MED | SUM | O |
| *Harpalus subcylindricus* | 1 | M | PHY | MED | SUM | O |
| *Harpalus tardus* | 68 | M | PHY | MED | SUM | O |
| *Limodromus assimilis* | 240 | M | ZOO | MED | SUM | F |
| *Limodromus krynickii* | 34 | M | ZOO | MED | SUM | F |
| *Metallina lampros* | 148 | M | ZOO | SMA | SUM | MH |
| *Microlestes minutulus* | 49 | M | ZOO | SMA | SUM | O |
| *Nebria brevicollis* | 1 | B | ZOO | MED | WIN | F |
| *Notiophilus substriatus* | 1 | M | ZOO | SMA | SUM | O |
| *Ophonus diffinis* | 4 | M | PHY | MED | WIN | O |
| *Ophonus puncticeps* | 1 | M | PHY | MED | WIN | O |
| *Parophonus hirsutulus* | 4 | M | PHY | MED | SUM | O |
| *Patrobus atrorufus* | 27 | B | ZOO | MED | WIN | F |
| *Poecilus cupreus* | 5 | M | OM | MED | SUM | O |
| *Poecilus versicolor* | 1206 | M | ZOO | MED | SUM | O |
| *Pseudoophonus griseus* | 11 | M | ZOO | MED | WIN | O |
| *Pseudoophonus rufipes* | 317 | M | OM | MED | WIN | O |
| *Pterostichus anthracinus* | 24 | D | ZOO | MED | ANN | W |
| *Pterostichus macer* | 1 | M | ZOO | MED | WIN | O |
| *Pterostichus melanarius* | 319 | B | ZOO | SMA | WIN | F |
| *Pterostichus niger* | 50 | M | ZOO | LAR | WIN | F |
| *Pterostichus strenuus* | 26 | M | ZOO | MED | ANN | F |
| *Pterostichus vernalis* | 8 | M | ZOO | MED | ANN | F |
| *Stomis pumicatus* | 42 | B | ZOO | MED | SUM | F |
| *Syntomus truncatellus* | 5 | D | ZOO | SMA | SUM | O |
| *Synuchus vivalis* | 113 | D | PHY | MED | WIN | F |
| *Trechus quadristriatus* | 16 | M | ZOO | SMA | WIN | MH |

# Table S2. Results of the Kruskal-Wallis test and the Mann-Whitney post hoc test applied for the comparison among transects (A, B, C) and habitats (Forest, Forest edge, Grassland, Tree row edge, Tree row) of carabids richness and abundance grouped into six ecological subsets: B = brachypetrous; M = macropterous; ZOO = zoophagous; PHY= phytophagous; MED = medium; LAR = large.

| Categories | Subset | N species | | | N inidividuals | | |
| --- | --- | --- | --- | --- | --- | --- | --- |
|  |  | *χ²* | *p* | *Post-hoc test* | *χ²* | *p* | *Post-hoc test* |
| Transects | ZOO | 4.991 | 0.082 | **-** | 11.416 | **0.003** | A > B, C |
|  | PHY | 4.520 | 0.104 | **-** | 4.112 | 0.128 | - |
|  | M | 5.375 | 0.068 | **-** | 16.634 | **0.000** | A > B, C |
|  | B | 4.117 | 0.128 | **-** | 4.518 | 0.105 | - |
|  | MED | 6.649 | **0.036** | A > C | 15.549 | **0.000** | A > B, C |
|  | LAR | 2.057 | 0.358 | **-** | 2.514 | 0.285 | - |
| Habitat | ZOO | 20.412 | **0.000** | Forest > Grassland | 10.329 | **0.035** | Forest > Grassland |
|  | PHY | 18.211 | **0.001** | Grassland > Forest | 9.192 | 0.056 | - |
|  | M | 0.204 | 0.995 | **-** | 1.607 | 0.808 | - |
|  | B | 12.786 | **0.012** | Forest > Grassland | 23.648 | **0.000** | Forest > Grassland |
|  | MED | 1.077 | 0.898 | **-** | 3.521 | 0.475 | - |
|  | LAR | 23.466 | **0.000** | Forest > Tree row, Grassland | 25.925 | **0.000** | Forest > Grassland |

**Table S3** Results from PERMANOVA for differences in carabid species composition among the factors “transect” (A,B,C) and "habitat" (tree row, tree row edge, grassland, forest edge and forest), and their interactions based on a Bray-Curtis resemblance matrix with *P*-values obtained by

9999 permutation. The analysis was performed on the entire sample of species and on five subsets (ZOO = zoophagous, PHY = phytophagous, B = brachypetrous, M = macropterous, MED = medium species) built according to the ecological characteristics (see the text for more details).

The p-value tests on the Monte Carlo computational method have been reported to compensate for the low number of permutations available for some comparisons

| Subset | Transect | | | Habitat | | | Transect × Habitat | | | |
| --- | --- | --- | --- | --- | --- | --- | --- | --- | --- | --- |
|  | *df* | *Pseudo-F* | *p (MC)* | *df* | *Pseudo-F* | *p (MC)* | | *df* | *Pseudo-F* | *p (MC)* |
| ZOO | 2 | 0.818 | 0.595 | 4 | 4.942 | **0.001** | | 8 | 1.086 | 0.384 |
| PHY | 2 | 0.646 | 0.685 | 4 | 1.520 | 0.157 | | 8 | 0.720 | 0.793 |
| B | 2 | 0.445 | 0.859 | 4 | 5.107 | **0.001** | | 8 | 0.701 | 0.783 |
| M | 2 | 0.669 | 0.767 | 4 | 3.879 | **0.001** | | 8 | 1.173 | 0.262 |
| MED | 2 | 1.160 | 0.318 | 4 | 5.248 | **0.001** | | 8 | 1.328 | 0.116 |

**Table S4** Results from PERMANOVA pairwise tests for differences in carabid species composition between pairs of habitats for the subsets: ZOO = zoophagous, B = brachypetrous, M = macropterous, MED = medium species. The p-value tests on the Monte Carlo computational method have been reported to compensate for the low number of permutations available for some comparisons

| Biotops | ZOO | | B | | M | | MED | |
| --- | --- | --- | --- | --- | --- | --- | --- | --- |
|  | *t* | *p (MC)* | *t* | *p (MC)* | *t* | *p (MC)* | *t* | *p (MC)* |
| *Tree row* | 2.189 | **0,004** | 2.286 | **0.013** | 1.868 | **0.010** | 1.963 | **0.011** |
| *Tree row, forest* | 1.752 | **0.045** | 2.516 | **0.013** | 1.460 | 0.106 | 1.477 | 0.131 |
| *Tree row edge, grassland* | 1.288 | 0.153 | 1.774 | 0.062 | 0.931 | 0.468 | 1.267 | 0.154 |
| *Tree row edge, Forest* | 2.778 | **0.005** | 3.063 | **0.005** | 2.551 | **0.009** | 3.155 | **0.004** |
| *Grassland, forest edge* | 1.711 | **0.032** | 1.821 | **0.043** | 1.244 | 0.186 | 1.366 | 0.125 |
| *Grassland, Forest* | 3.706 | **0.001** | 3.635 | **0.001** | 3.282 | **0.001** | 3.882 | **0.001** |
| *Forest edge, Forest* | 1.844 | **0.034** | 1.578 | 0.106 | 2.217 | **0.013** | 2.032 | **0.021** |

**Table S5.** Effect of the environmental variables on the species richness present in the study area obtained by means of Multi Model Inference. The table shows the estimates of the standardized mean regression coefficients (β) and the relative importance (Ri) of each environmental variable in each subsets (B = brachypetrous, M = macropterous, D = dimorphic, ZOO = zoophagous, OM = omnivorous, PHY= phytophagous, MED = medium and LAR = large). The variables found to be not significant in each subset are not shown in the table. The relative importance (Ri) was calculated as the importance (sum of the "Akaike weights" of all models) of a given variable divided by the sum of the importance of all the variables for each subset of data. The dashes indicate that the term considered does not appear in the best model set. Variables whose β confidence interval does not include 0 can be considered to have a significant effect (values ​​expressed in bold).

| Subset | Humidity | | Simple crops | | Riparian vegetation | | Soparse forest | | “Bosco Negri” forest | | Temperature | |
| --- | --- | --- | --- | --- | --- | --- | --- | --- | --- | --- | --- | --- |
|  | β | Ri | β | Ri | β | Ri | β | Ri | β | Ri | β | Ri |
| B | **1.339** | **0.622** | - | - | - | - | -0.006 | 0.111 | -0.011 | 0.155 | - | - |
| M | 0.420 | 0.136 | **-0.013** | **0.621** | -0.013 | 0.152 | - | - | -0.007 | 0.091 | - | - |
| D | 0.153 | 0.173 | **-0.004** | **0.162** | **-0.011** | **0.260** | -0.007 | 0.206 | 0.004 | 0.153 | - | - |
| ZOO | **2.581** | **0.326** | - | - | **-0.038** | **0.184** | **-0.020** | **0.184** | -0.019 | 0.097 | - | - |
| OMN | - | - | **-0.007** | **0.435** | -0.010 | 0.261 | 0.003 | 0.046 | - | - | - | - |
| PHY | **-1.525** | **0.304** | **-0.017** | **0.304** | 0.015 | 0.037 | 0.000 | 0.000 | - | - | 0.230 | 0.076 |
| LAR | **0.722** | **0.307** | 0.004 | 0.047 | **-0.015** | **0.307** | -0.005 | 0.077 | -0.007 | 0.051 | -0.155 | 0.211 |
| MED | **1.150** | **0.232** | **-0.021** | **0.232** | -0.018 | 0.160 | -0.010 | 0.065 | -0.012 | 0.080 | **0.259** | **0.232** |

**Table S6.** Effect of the environmental variables on the abundance of carabid species present in the study area obtained by means of Multi Model Inference. The table shows the estimates of the standardized mean regression coefficients (β) and the relative importance (Ri) of each environmental variable in each subsets (B = brachypetrous, M = macropterous, D = dimorphic, ZOO = zoophagous, OM = omnivorous, PHY= phytophagous, MED = medium and LAR = large). The variables found to be not significant in each subset are not shown in the table. The relative importance (Ri) was calculated as the importance (sum of the "Akaike weights" of all models) of a given variable divided by the sum of the importance of all the variables for each subset of data. The dashes indicate that the term considered does not appear in the best model set. Variables whose β confidence interval does not include 0 can be considered to have a significant effect (values ​​expressed in bold).

| Subset | Humidity | | Simple crops | | Dense forest | | Vegetation cover | | Sparse forest | | Riparian vegetation | |
| --- | --- | --- | --- | --- | --- | --- | --- | --- | --- | --- | --- | --- |
|  | β | Ri | β | Ri | β | Ri | β | Ri | β | Ri | β | Ri |
| B | **28.603** | **0.251** | 0.242 | 0.166 | - | - | **-19.034** | **0.251** | -0.364 | 0.206 | - | - |
| M | **30.713** | **0.279** | **-0.548** | **0.279** | **-0.418** | **0.279** | 24.657 | 0.162 | - | - | - | - |
| D | - | - | -0.056 | 0.310 | - | - | 0.927 | 0.028 | 0.077 | 0.177 | **-0.067** | **0.198** |
| ZOO | **54.860** | **0.293** | -0.369 | 0.122 | **-0.388** | **0.225** | - | - | **-0.621** | **0.225** | - | - |
| OMN | **9.804** | **0.261** | **-0.117** | **0.261** | **-0.101** | **0.120** | **10.459** | **0.261** | - | - | -0.169 | 0.097 |
| PHY | **-4.448** | **0.432** | **-0.050** | **0.432** | - | - | - | - | - | - | -0.040 | 0.136 |
| LAR | **25.181** | **0.398** | 0.046 | 0.083 | 0.079 | 0.122 | **-11.320** | **0.398** | - | - | - | - |
| MED | **29.506** | **0.250** | **-0.515** | **0.176** | **-0.454** | **0.200** | 15.714 | 0.050 | -0.659 | 0.154 | - | - |
